# Delayed diagnosis and increased mortality risk: Assessing the effects of the COVID-19 pandemic on breast cancer recurrence

**DOI:** 10.1016/j.clinsp.2024.100340

**Published:** 2024-03-02

**Authors:** Lindson Mühlmann, Franklin Fernandes Pimentel, Daniel Guimarães Tiezzi, Hélio Humberto Angotti Carrara, Jurandyr Moreira de Andrade, Francisco José Candido dos Reis

**Affiliations:** Department of Gynecology and Obstetrics, Faculdade de Medicina de Ribeirão Preto, Universidade de São Paulo, Ribeirão Preto, SP, Brazil

**Keywords:** Breast neoplasms, COVID-19, Disease recurrence

## Abstract

**Objectives:**

The COVID-19 pandemic has had a significant global impact since its declaration in March 2020. The COVID-19 pandemic has disproportionately impacted cancer patients, particularly those with breast cancer. This study aims to analyze the effects of the pandemic on women diagnosed with breast cancer recurrence.

**Methods:**

A cohort study was conducted at a tertiary public hospital in São Paulo State, Brazil. Data were collected from electronic records. Patients diagnosed with breast cancer and experiencing recurrence between January 2011 and March 2022 were included. Survival analysis was performed using the Kaplan-Meier estimator and Cox regression.

**Results:**

The study included 187 patients, 45 in the pandemic group (recurrence after March 23, 2020) and 142 in the pre-pandemic group. Distant recurrences were more frequent in both groups (pre-pandemic: 62.7 %, pandemic: 75.5 %). Compared to the pre-pandemic group (1.8 years), the pandemic group experienced a longer mean time to recurrence detection (2.9 years) and significantly decreased median survival (9 months vs. 22 months). The Cox regression analysis confirmed an increased risk of death for women diagnosed with breast cancer recurrence during the pandemic period (HR = 1.92, 95 % CI 1.19‒3.12).

**Conclusion:**

The present study is among the first to investigate the pandemic's specific effects on breast cancer recurrence, revealing concerning delays in detection and a decrease in survival rates. Prompt diagnosis, timely treatment initiation, and comprehensive support are crucial during public health crises. These findings urge healthcare systems to prioritize tailored care for breast cancer patients during pandemics.

## Highlights

• COVID-19 profoundly affected global healthcare including breast cancer management.
• The pandemic delayed recurrence diagnosis and increased post-recurrence mortality.
• Strategies for cancer patients' follow-up during health crises need improvements.

## Introduction

The COVID-19 pandemic, caused by the spread of the SARS-CoV-2 virus, has had a significant global impact since its declaration as a pandemic by the World Health Organization on March 11, 2020.^1^^,^^2^ Patients with cancer who contracted COVID-19 were at a higher risk of severe outcomes.^3^ Breast cancer is the most common cancer among women, with a substantial number of cases worldwide.^4^ During the pandemic, medical appointments for low-risk breast cancer patients in the post-treatment phase were deprioritized, while high-risk patients and those with genetic mutations received medium priority.^5^

Breast cancer recurrence can be detected through three primary means: (1) Patient presentation of signs/symptoms leading to medical consultation outside of scheduled appointments; (2) Signs/symptoms reported during routine check-ups, prompting further clinical investigation; (3) Absence of signs/symptoms, with recurrence identified through clinical examination during routine appointments.^6^ The patterns of detecting treatable breast cancer recurrence have shown a change over time. Earlier studies up to the year 2000 found that about 46 % of treatable recurrences were identified through routine clinical examination. However, more recent studies indicate a significant decrease in this detection method, with only approximately 15 % of treatable recurrences being detected through routine clinical examination.^7^ Prior research has emphasized the concern with understanding recurrence patterns and other negative impacts of the pandemic on breast cancer patients.^7^, ^8^, ^9^, ^10^, ^11^, ^12^

Building on these insights, this study aims to analyze how the COVID-19 pandemic has affected breast cancer recurrence detection and clinical outcomes. We compare several factors, including patient characteristics, follow-up duration upon recurrence, types of recurrence, and post-recurrence survival, between two groups: patients diagnosed with breast cancer recurrence during the pandemic and those diagnosed before it. Understanding these patterns is crucial to optimize cancer care during public health crises and potentially minimize negative impacts on patients with breast cancer.

## Material and methods

An observational, historical cohort study conducted at the Clinics Hospital of the Faculdade de Medicina de Ribeirão Preto, Universidade de São Paulo, investigated breast cancer recurrence patterns among women previously treated for the disease. This study aimed to assess the impact of exposure to the COVID-19 pandemic on recurrence patterns and survival outcomes. Data were collected from electronic medical records via REDCap electronic data capture forms, allowing secure monitoring by the research team.

The Research Ethics Committee of the Clinics Hospital of the Faculdade de Medicina de Ribeirão Preto, Universidade de São Paulo, granted ethical approval for the study under opinion 4.853.121.

Among 2,891 eligible records of patients with breast cancer and complete follow-up between January 2011 and March 2022, we identified 187 patients with recurrences. We excluded patients without recurrence, with de novo breast cancer metastasis at the diagnosis, those with a prior diagnosis of another cancer type, patients diagnosed before 2011, with non-epithelial breast tumors (e.g., phyllodes tumor, sarcoma, metaplastic carcinomas), and patients who didn't complete the initial treatment, focusing this analysis on female breast cancer recurrences and minimizing potential confounding factors.

Following the detection of metastatic recurrence, systemic treatment was selected based on local guidelines, considering the breast cancer subtype at presentation or the subtype identified from the metastasis biopsy, if feasible and clinically relevant. At this institution, during the analyzed period and considering drug affordability in the public health system, patients with metastatic triple-negative breast cancer had access to chemotherapy (paclitaxel, docetaxel, doxorubicin, capecitabine, vinorelbine, gemcitabine, carboplatin, cisplatin) for monotherapy or combination therapy as preferred by the attending physician in sequential lines. Hormone receptor-positive and HER2-negative metastatic patients were offered endocrine treatment if suitable, or chemotherapy (paclitaxel, docetaxel, capecitabine, vinorelbine, gemcitabine, doxorubicin) in sequential lines, if necessary, unless there were visceral crisis or endocrine resistance. Patients with HER2-positive breast cancer received trastuzumab and docetaxel (associated with pertuzumab since 2020 if there was visceral metastasis), followed by trastuzumab maintenance until progression, and subsequent chemotherapy or endocrine therapy adjustments were made accordingly.

Patients with locoregional recurrence underwent an evaluation to determine if they were candidates for surgery with curative intent. If surgery was feasible, adjuvant chemotherapy was typically administered for triple-negative or HER2-positive patients, and endocrine treatment was given to those who were ER+ HER2-negative. In cases where locoregional recurrence was unresectable, patients received treatment regimens for metastatic recurrence.

This study utilized several variables to explore breast cancer recurrence during the COVID-19 pandemic. These included: patient age at diagnosis, primary tumor characteristics (histology, grade, staging according to the 8th edition TNM system, molecular markers, and molecular classification), comprehensive treatment history, and follow-up data. Patients were divided into two groups based on recurrence detection: the pandemic group (after March 23, 2020) and the pre-pandemic group (before March 23, 2020). For continuous variables, we analyzed both the average and spread (standard deviation) in each group. Qualitative variables were presented as both total numbers and percentages of each category within each group.

Statistical analysis was conducted using RStudio (RStudio: Integrated Development for R, RStudio, Inc., Boston, MA; http://www.rstudio.com/). We employed Student's *t*-test to compare quantitative variables between groups, with consideration for variable distribution. For categorical variables, we utilized the Chi-Square test. Survival analysis was performed using the Kaplan-Meier product limit estimator and the Cox proportional hazards regression model. The observation period commenced on the date of recurrence diagnosis, with a maximum follow-up time of two years (right censoring).

## Results

**Fig. 1:**
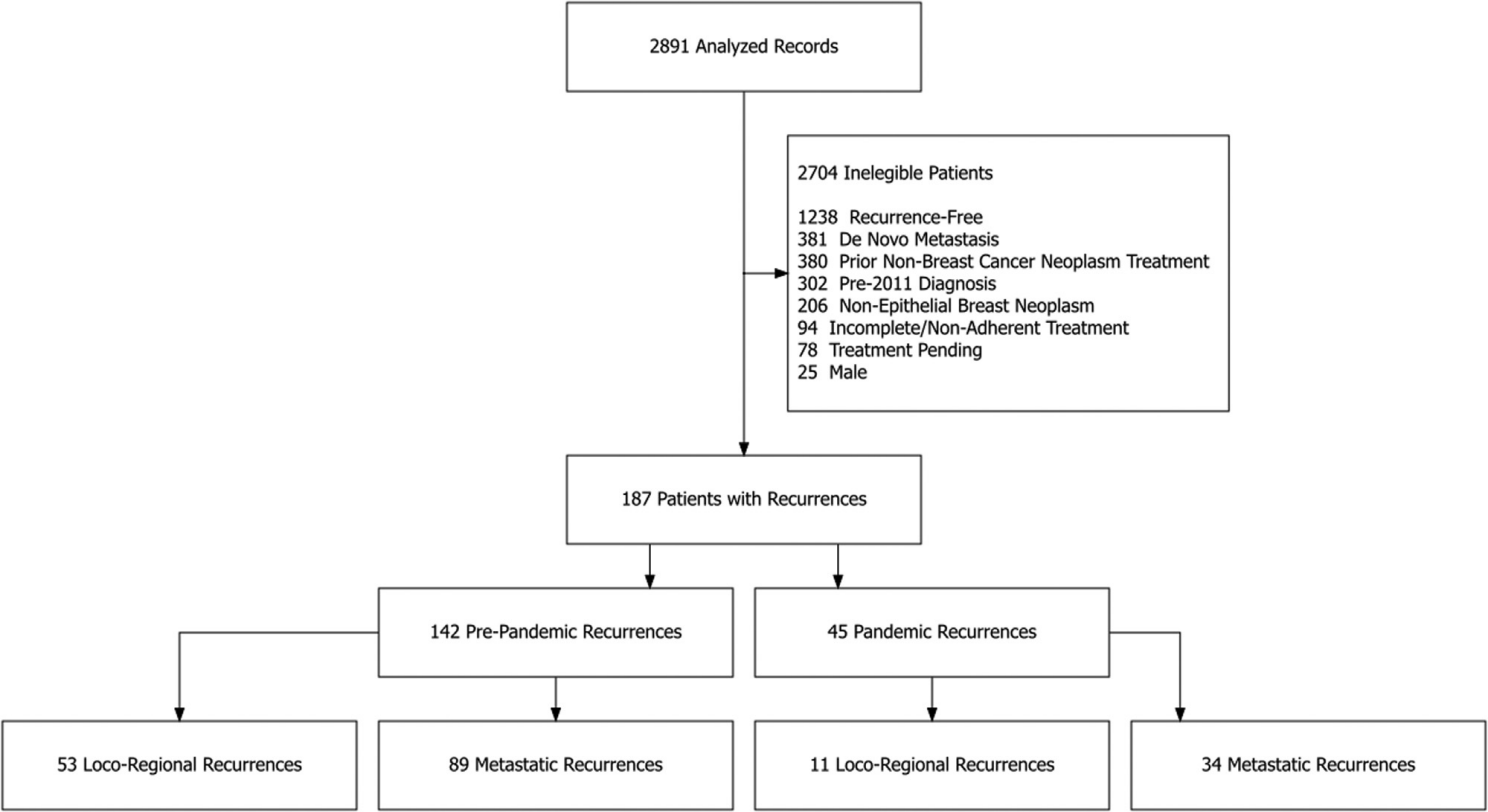
Distribution of breast cancer recurrence cases during the COVID-19 pandemic and pre-pandemic periods.

The study cohort included 187 women with breast cancer recurrence. Of these, 45 were diagnosed with recurrence after March 23, 2020, constituting the pandemic group, while the remaining 142 patients, diagnosed between January 1, 2011, and March 22, 2020, formed the pre-pandemic group as further detailed in Fig. 1.

Notably, the pandemic group exhibited a longer time interval between the beginning of follow-up and the date of recurrence detection, with a mean duration of 2.9 years (SD = 1.8), compared to the pre-pandemic group's mean of 1.8 years (SD = 1.5). There was a statistical difference between the groups (p < 0.001).

Table 1 presents the characteristics of the study participants. Distant recurrences were more frequent in both the pre-pandemic group, accounting for 62.7 %, and the pandemic group, with 75.5 %. The majority of patients in both groups received chemotherapy, with rates of 77.5 % in the pre-pandemic group and 82.2 % in the pandemic group.

**Table 1:** Characteristics of women with breast cancer recurrence during the COVID-19 pandemic and pre-pandemic period.

Variable	Pandemic	Pre-pandemic	p-value
Cases	n = 45	n = 142	
Age (years)	58.8 (13.5)	58.1 (14.6)	0.78
**Histological type**			
Invasive Ductal Carcinoma	44 (97.8 %)	131 (92.3 %)	0.39
Invasive Lobular Carcinoma	1 (2.2 %)	8 (5.6 %)	
Others	0	3 (2.1 %)	
**Tumor grade**			
I	8 (17.8 %)	27 (19 %)	0.80
II	25 (55.6 %)	84 (59.2 %)	
III	12 (26.7 %)	31 (21.8 %)	
**Anatomical staging**			
IA	4 (8.9 %)	9 (6.3 %)	0.89
IB	0	1(0,7 %)	
IIA	7 (15.6 %)	20 (14.1 %)	
IIB	7 (15.6 %)	30 (21.1 %)	
IIIA	11 (24.4 %)	33 (23.2 %)	
IIIB	15 (33.3 %)	41 (28.9 %)	
IIIC	1 (2.2 %)	8 (5.6 %)	
**Prognostic staging**			
IA	4 (8.9 %)	10 (7 %)	0.81
IB	5 (11.1 %)	20 (14.1 %)	
IIA	6 (13.3 %)	27 (19 %)	
IIB	5 (11.1 %)	8 (5.6 %)	
IIIA	10 (22.2 %)	26 (18.3 %)	
IIIB	12 (20.7 %)	43 (30.3 %)	
IIIC	3 (6.7 %)	8 (5.6 %)	
**Estrogen receptor**			
Negative	16 (35.6 %)	62 (43.7 %)	0.43
Positive	29 (64.4 %)	80 (56.3 %)	
**Progesterone receptor**			
Negative	26 (57.8 %)	75 (52.8 %)	0.68
Positive	19 (42.2 %)	67 (47.2 %)	
**HER2 Immunohistochemistry**			
0	18 (40 %)	51 (35.9 %)	0.90
1+	12 (26.7 %)	46 (32.4 %)	
2+	5 (11.1 %)	16 (11.3 %)	
3+	10 (22.2 %)	29 (20.4 %)	
**HER2 (status)**			
Negative	33 (73.3 %)	109 (76.8 %)	0.79
Positive	12 (26.7 %)	33 (23.2 %)	
**Subtype**			0.39
HR+ HER2-	25 (55 %)	67 (47 %)	
HR+ HER2+	4 (9 %)	15 (11 %)	
HR- HER2+	8 (18 %)	18 (13 %)	
HR- HER2-	8 (18 %)	42 (29 %)	
**Chemotherapy**			
Neoadjuvant	29 (64.4 %)	70 (49.3 %)	0.19
Adjuvant	8 (17.8 %)	40 (28.2 %)	
Not received	8 (17.8 %)	32 (22.5 %)	
**Hormone therapy**			
No	16 (35.6 %)	66 (46.5 %)	0.26
Yes	29 (64.4 %)	76 (53.5 %)	
**Radiotherapy**			
No	10 (22.2 %)	26 (18.3 %)	0.72
Yes	35 (77.8 %)	116 (81.7 %)	

**Fig. 2:**
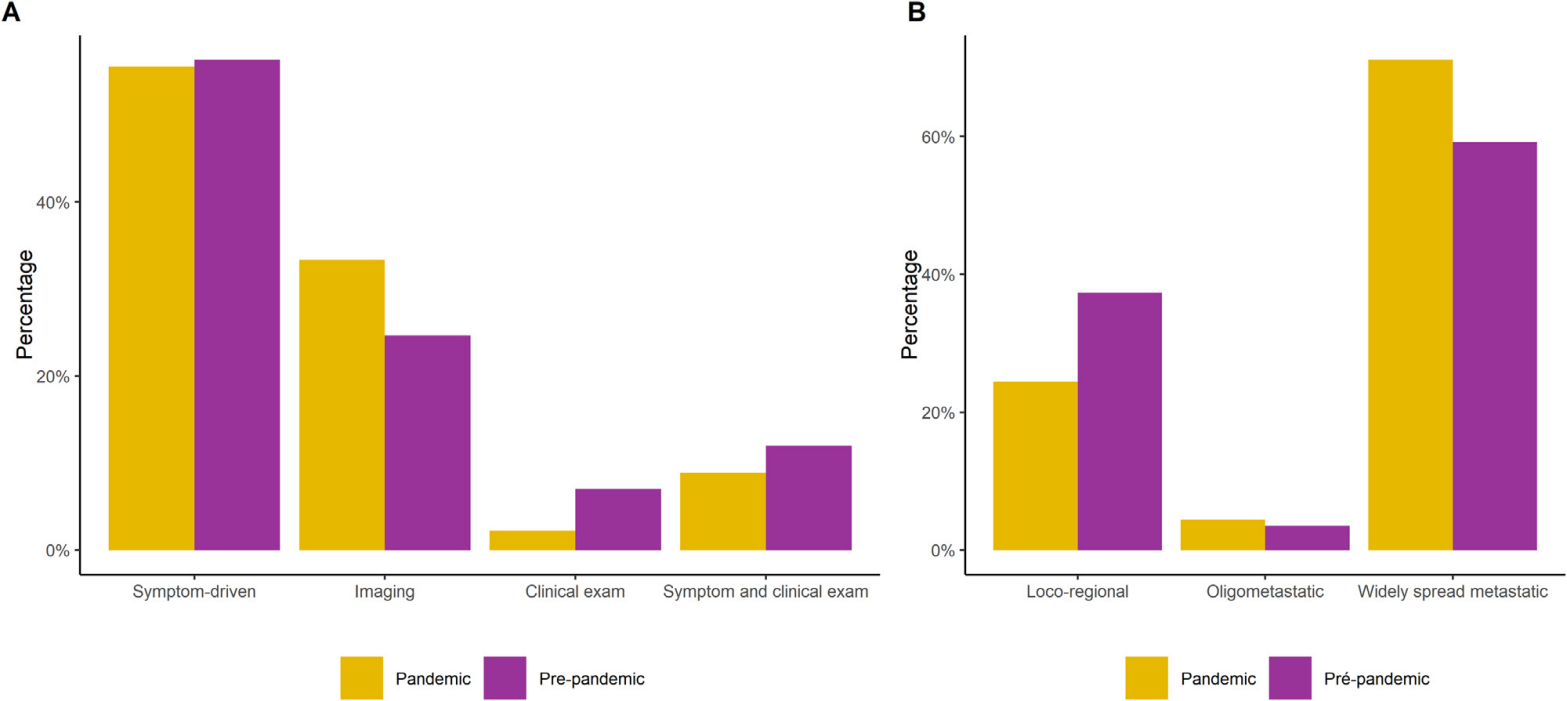
Recurrence characteristics: (A) Modalities for detecting breast cancer recurrences in the COVID-19 pandemic versus pre-pandemic periods. (B) Distribution of recurrence sites in the pandemic and pre-pandemic periods.

Local-only recurrences were observed in 37 % of patients before the pandemic and 24 % during the pandemic. Notably, during the pandemic, 2 % of patients had a recurrence detected solely through physical exams, without presenting any previous symptoms, whereas in the pre-pandemic period, this proportion was 7 %. Clinical symptoms remained the primary reason for diagnosing recurrence in both periods, accounting for 56 % of cases. Detection through imaging was the second most common method, with rates of 25 % in the pre-pandemic group and 33 % in the pandemic group, particularly in asymptomatic patients (Fig. 2).

**Fig. 3:**
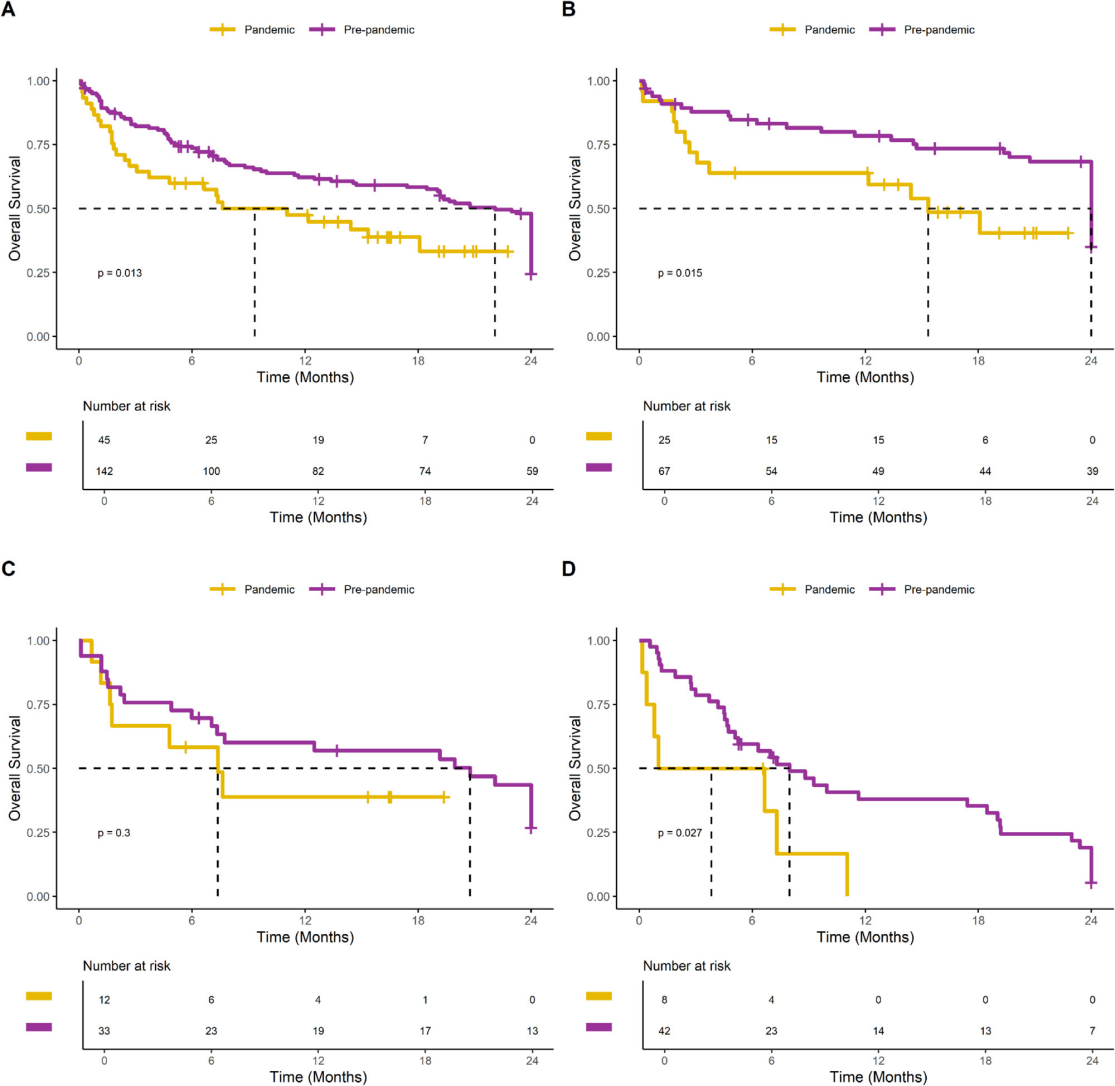
Survival analysis of women with breast cancer recurrence in the COVID-19 pandemic and pre-pandemic groups with 24 months of follow-up. (A) All patients. (B) Patients with luminal-like HER2-negative tumors. (C) Patients with HER2-positive tumors. (D) Patients with triple negative tumors.

The Kaplan-Meier analysis demonstrated a significant decrease in survival in the pandemic group compared to the pre-pandemic group (median 9 months versus 22 months, log-rank; p = 0.013). This negative impact of the pandemic period on survival after breast cancer recurrence was observed across all tumor subtypes, as illustrated in Fig. 3. The Cox regression analysis, presented in Table 2, further confirmed that the pandemic period was associated with an increased risk of death after breast cancer recurrence, with a hazard ratio of 1.92 (95 % CI 1.19‒3.12).

**Table 2:** Cox regression analysis for overall survival in women with breast cancer recurrence during the COVID-19 pandemic and pre-pandemic period.

Variable	HR	95 % Cl	p-value
**Groups**			
Pre-pandemic	Ref		
Pandemic	1.92	(1.19‒3.12)	0.008
Age	1.02	(1.01‒1.03)	0.01
Interval between follow-up and recurrence	0.99	(0.88‒1.12)	0.89
**Histological type**			
Invasive Ductal Carcinoma	Ref		
Invasive Lobular Carcinoma	1.5	(0.62‒3.58)	0.37
Others	0.33	(0.04‒2.73)	0.30
**Anatomical staging**			
IA	Ref		
IB	0.38	(0.05‒3.25)	0.38
IIA	0.82	(0.16‒4.2)	0.82
IIB	0.86	(0.2‒3.7)	0.84
IIIA	0.7	(0.19‒2.65)	0.60
IIIB	0.82	(0.28‒2.41)	0.71
IIIC	0.5	(0.13‒1.94)	0.32
**Prognostic staging**			
IA	Ref		
IB	0.48	(0.11‒2.1)	0.33
IIA	0.57	(0.16‒2.01)	0.38
IIB	0.36	(0.09‒1.52)	0.16
IIIA	0.85	(0.32‒2.25)	0.75
IIIB	0.97	(0.44‒2.12)	0.93
**Tumor grade**			
I	Ref		
II	0.84	(0.48‒1.45)	0.53
III	1.87	(0.93‒3.78)	0.08
**Estrogen receptor**			
Positive	Ref		
Negative	1.95	(1.12‒3.4)	0.02
**HER2 (status)**			
Negative	Ref		
Positive	0.71	(0.42‒1.2)	0.27

## Discussion

The impact of the COVID-19 pandemic on breast cancer recurrence is a major concern. The present study investigates this critical issue and provides substantial evidence for its negative influence on follow-up and outcomes for women with breast cancer. These findings reveal a worrying trend: the pandemic significantly delayed recurrence diagnosis and was associated with an increased risk of death after recurrence. These delays in diagnosis and subsequent treatment, a phenomenon confirmed by other studies, raise considerable alarm about the potential impact on patient survival.^10^

One of the notable effects of the pandemic was the increase in time for the detection of breast cancer recurrences. This delay can be attributed to various factors, including the reduction in hospital visits and modifications in the priority of face-to-face consultations, such as those for breast cancer follow-up. As the pandemic placed a strain on healthcare resources, medical facilities had to prioritize urgent cases, resulting in the deprioritization of routine and non-urgent visits, including those related to breast cancer surveillance.^13^, ^14^, ^15^

Furthermore, the disruptions caused by the pandemic led to delays in other essential services, such as mammography. Public health measures, lockdowns, and the temporary closure or reduction of screening programs contributed to the decreased availability of mammographic services. The reduced access to these diagnostic procedures likely hindered the early detection of breast cancer recurrence, resulting in delayed diagnosis and potentially more advanced disease at the time of detection.^16^^,^^17^

We observed an impact on the survival of patients whose recurrence was diagnosed after March 23, 2020, despite the continuity of treatment for women with breast cancer in the studied institution during the pandemic. The pandemic's influence extended beyond treatment interruptions, affecting multiple variables. These variables include the interval between symptoms and the diagnosis of recurrence, treatment choice, acceptance, adherence, time to initiate treatment, management of treatment-related toxicities, bed shortages, and concerns about COVID-19 infection. The magnitude of the risk of mortality associated with the pandemic period suggests that the pandemic's effect on breast cancer outcomes might be higher than expected based on early reports.^18^ Some studies already explain these effects based on delays or non-attendance at appointments^11^ or even the detection of advanced-stage breast cancer for patients who were diagnosed during the pandemic.^8^ The present data indicate that survival after breast cancer recurrence is influenced by various factors, including the type and extent of the recurrence, estrogen receptor status of the primary tumor, and the age of the patient, which aligns with previous findings.^19^, ^20^, ^21^

It is essential to acknowledge that this study has certain limitations. The retrospective design may introduce a risk of selection bias due to the potential for missing data. Further research, including studies with larger sample sizes and multicenter collaborations, is needed to enhance the generalizability of the present findings and provide a more comprehensive understanding of the impact of the COVID-19 pandemic on breast cancer recurrence outcomes. Furthermore, this study was conducted within the context of the Brazilian health system, which provides comprehensive coverage and medical assistance to all Brazilian citizens. Therefore, the results of this study may not be generalizable to other countries with different healthcare systems and resource allocations. The impact of the pandemic on breast cancer outcomes may vary depending on the healthcare infrastructure, access to resources, and healthcare policies in different countries. Despite these limitations, the present study contributes valuable insights into the effects of the COVID-19 pandemic on women with breast cancer recurrence.

While physical distancing during the pandemic may have been an initial concern, this study reveals that reliance on solely physical examinations for recurrence detection has decreased. This aligns with the improvements in complementary imaging exams and advancements in healthcare technologies since the 2000s.^7^ However, the present findings also highlight the crucial role of patient awareness and self-detection, present in both pandemic and pre-pandemic groups. This underscores the importance of intensified public information campaigns and encouraging patients to seek immediate medical attention upon noticing any abnormalities.

This study's identification of the COVID-19 pandemic's detrimental impact on survival and healthcare access underscores the urgent need for adaptation and innovative strategies to optimize breast cancer care during public health crises. By illuminating the medium and long-term challenges faced by patients, this research can inform interventions to mitigate negative effects, particularly on survival timelines. This includes emphasizing the importance of thorough routine consultations, prompt action upon noticing any abnormalities, and proactive utilization of complementary exams. These recommendations hold particular weight for patients experiencing recurrent breast cancer within the pandemic timeframe.

## Conclusion

The present study provides compelling evidence for the COVID-19 pandemic's detrimental impact on breast cancer recurrence management. The authors observed significantly extended delays in recurrence diagnosis and a heightened risk of post-recurrence mortality, highlighting the pandemic's widespread influence beyond treatment disruptions. These stark findings underscore the urgent need to adjust these strategies, prioritize prompt diagnosis and timely treatment initiation, and ensure comprehensive support for women with recurrent breast cancer navigating public health crises. Further research is needed to refine targeted interventions and strengthen support systems, paving the way for improved care during future pandemics.

## Funding

This project was supported by the Coordenação de Aperfeiçoamento de Pessoal de Nível Superior (CAPES) - Program CAPES EPIDEMIAS [grant n° 88887.506852/2020-00]. L Mühlmann was funded by the Coordenação de Aperfeiçoamento de Pessoal de Nível Superior (CAPES) [grant n° 88887.517592/2020-00]. F. J. Candido dos Reis was funded by the Council for Scientific and Technological Development (CNPq) [grant n° 310262/2021-6].

## Data availability

Data generated during the study may be requested from the corresponding author.

## CRediT authorship contribution statement

**Lindson Mühlmann:** Conceptualization, Visualization, Data curation, Formal analysis, Writing – review & editing. **Franklin Fernandes Pimentel:** Conceptualization, Visualization, Writing – review & editing. **Daniel Guimarães Tiezzi:** Writing – review & editing. **Hélio Humberto Angotti Carrara:** Writing – review & editing. **Jurandyr Moreira de Andrade:** Writing – review & editing. **Francisco José Candido dos Reis:** Conceptualization, Visualization, Formal analysis, Writing – review & editing.

## Conflicts of interest

The authors declare no conflicts of interest.
